# Effects of Four-Week Kayak Training on Three-Dimensional Paddling Kinetics, Body Kinematics, and Electromyography Activity in a Novice Paddler: A Case Study

**DOI:** 10.3389/fspor.2021.694989

**Published:** 2021-07-27

**Authors:** Ryuta Kinugasa, Shimpei Kubo, Keiko Endo

**Affiliations:** Faculty of Human Sciences, Kanagawa University, Yokohama, Japan

**Keywords:** average impulse, beginner, biomechanics, kayak ergometer, paddling, surfski

## Abstract

From a biomechanical viewpoint, no longitudinal quantitative studies have been conducted on inexperienced paddlers. The present study aimed to investigate changes in three-dimensional paddling kinetics and kinematics, whole-body kinematics, and muscle activity with four-week on-water kayak training in a novice paddler. The participant practiced kayak paddling on river for four weeks. Before and after training, paddling kinetics and kinematics, body kinematics, and electromyography (EMG) activity were measured using a kayak ergometer. After the four-week training, the time required for on-water paddling for 270 m was reduced by 7.3% from pre to post training, while the average impulse in the x-direction significantly (*P* < 0.001, partial eta squared [η^2^] = 0.82) increased from 71.9 ± 1.9 to 91.1 ± 5.4 N kg^−1^ s^−1^. Furthermore, with training, the stroke rate and stroke length in the x-direction significantly (*P* < 0.001, partial η^2^ = 0.80 and 0.79, respectively) increased from 62.8 ± 1.2 to 81.0 ± 2.9 spm and from 1.53 ± 0.04 to 1.71 ± 0.02 m, respectively. After training, the transition time significantly (*P* < 0.001, partial η^2^ = 0.32) decreased (from 0.04 ± 0.01 to 0.01 ± 0.01 s), and there was an increase in paddle catch position (from −0.88 ± 0.01 to −1.04 ± 0.03 m). The pull time was not significantly changed (*P* = 0.077, partial η^2^ = 0.08) because of the increasing stroke length after training, meaning that substantial pull time, which defined as pull time relative to the stroke displacement, was shorter in post-training than in pre-training. The relative change in average impulse in the x-direction with training was significantly (*r* = 0.857, *P* = 0.014) correlated with that of vastus lateralis EMG. These results indicated that after four-week kayak training of the novice paddler, the key mechanism underlying time reduction to perform on-water paddling for 270 m was associated with (1) increased average impulse along the propulsive direction caused by an increase in vastus lateralis EMG and (2) a higher stroke rate, which was attributed to a reduction in the pull and transition times.

## Introduction

Mechanical loading caused by inappropriate paddling skills is an important factor behind pain development among kayak and canoe paddlers (Walsh, 1989). According to questionnaire results, more than half of flat-water kayak paddlers experienced shoulder injuries (Walsh, 1989; Edwards, 1993; Toohey et al., 2019). Indeed, injuries typically occur in the upper body regions, with shoulder, thoracic, lumbar, and pelvic/hip injuries accounting for 27%, 13%, 12%, and 10%, respectively (Toohey et al., 2019). Therefore, in order to prevent chronic injuries associated with the practices of kayak paddlers, experienced paddlers should possess the ability to identify and correct problematic paddling skills, whereas beginners should learn proper paddling skills.

Paddling skills are evaluated using biomechanical parameters. Several biomechanical studies have focused on the paddling skills of experienced paddlers, including international-level paddlers. In contrast, studies on novice paddlers are very limited. With a cross-sectional design, two research groups have included novice paddlers in their studies. Sanders and Kendal (1992) examined paddlers ranging in experience from novice to elite to determine factors associated with superior performance; they reported that the on-water paddling performance of novice paddlers was associated with a low stroke frequency, which was attributed to the maximization of both pull and glide times. On the other hand, Limonta et al. (2010) conducted a three-dimensional motion analysis to measure the angular displacement of the elbows, knees, pelvis, and torso among elite, intermediate-level, and novice paddlers and reported that the novice paddlers displayed a significantly lower stroke length than the international- and intermediate-level paddlers, which could be explained by the insufficient rotations of the pelvis and torso.

Only one longitudinal study was conducted on novice paddlers. Yoshio et al. (1974) visually compared unprocessed data and showed that the muscle activity patterns of the upper limbs were not different between skilled and inexperienced paddlers and that four-week practice had no effect on the muscle activity in beginners. To understand the paddling skills of novice paddlers, it is necessary to at least quantify their whole-body kinematics, as well as the force generated in the paddle shaft and the muscle activity while paddling. Therefore, the present study aimed to investigate the effects of four-week on-water kayak training on whole-body kinematics, paddling kinetics and kinematics, and muscle activity in a novice paddler. The training period was based on a previous study (Sakadjian et al., 2014), which suggested that four weeks were sufficient to change performance and acquire sports skills. As greater pelvic and torso rotations are acquired for the transition from a beginner to an intermediate-level paddler (Limonta et al., 2010), we hypothesized that four-week kayak training for a novice paddler would lead to increased pelvic and torso rotations, which would facilitate an increase in stroke length.

## Materials and Methods

### Participant

A 43-year-old novice male paddler (height, 167.0 cm; weight, 59.7 kg) volunteered to participate in this study. He provided written informed consent before participation. This study was conducted in accordance with the tenets of the Declaration of Helsinki and was approved by the local ethics committee.

### Training

Training was conducted for four weeks (3–5 days per week, 9–95 min per day) and consisted of kayak paddling practice on the river using a surfski apparatus (Epic V5; Epic Kayaks, TN, USA). The training program is summarized in Supplementary Table 1.

### Experimental Design

Two tests were performed before and after training. With respect to the on-water test, the participant was required to perform all-out paddling for 270 m on the river. The paddling on-ergometer test with a kayak ergometer (K1 Ergo; Australian Sports Commission, Canberra, Australia) lasted for ~20 s. The participant was capable of completing at least seven strokes at the following stroke rates: 56 strokes per min (spm) (defined as slow stroke rate), 69 spm (defined as medium stroke rate), and maximal stroke rate (defined as the rate at which the paddler needed to paddle to the maximum possible extent). The order of stroke rates was randomized. A recovery time of 1 min was allowed between the trials.

### Measurements

#### On-Water Test

The all-out paddling time was determined by requesting the paddler to complete a specified distance of 270 m and timed with a stopwatch by one of the investigators.

#### On-Ergometer Test

A strain gauge load cell (LUR-A-1KNSA1; Kyowa Electronic Instruments, Tokyo, Japan) was placed between the rope from the ergometer flywheel and the right end of the paddle shaft. The force was collected during paddling at a sampling rate of 1500 Hz. We performed the calibration statically by loading the calibrated weights.

A motion-capture system (Raptor-12; Motion Analysis, CA, USA) was used to measure the three-dimensional coordinates of reflective markers during the paddling on-ergometer test. The placement of reflective markers is described in Supplemental Materials. We defined the x-, y-, and z-axes in the global coordinate system as the posterior (+)/anterior (–), lateral (+)/medial (–), and superior (+)/inferior (–) directions, respectively. The frame rate of the motion-capture system was 150 Hz.

Electromyography (EMG) recordings were captured from seven muscles (biceps brachii, triceps brachii, anterior deltoid, trapezius, latissimus dorsi, vastus lateralis, musculus obliquus externus abdominis) on the dominant side of the body using standard wireless electrodes (Trigno; Delsys, MA, USA). The placement and preparation of electrodes are detailed in the Methods section of Supplemental Materials.

### Data Analysis

Details of data analysis are described in the Methods section of Supplemental Materials. One stroke cycle was divided into three phases: pull, transition, and return. The pull phase began from the minimum value of the x-coordinate of the right edge marker of the paddle shaft to just before the transition phase (Supplementary Figure 1). The transition phase began from the maximum value of the y-coordinate of the right edge marker of the paddle shaft to the maximum value of the y-coordinate of the left edge marker of the paddle shaft. The return phase began from the maximum value of the x-coordinate of the right edge marker of the paddle shaft to just before the pull phase in the next cycle. The force vector was calculated from the load cell and motion-capture system and was subsequently separated into the force in each axis in the global coordinate system. The peak force and impulse in each axis were calculated as the peak value and integration of force during the pull phase, respectively. The average impulse was calculated as the impulse divided by the time of one stroke cycle. We calculated the duration of each phase and stroke rate. The stroke length was defined as the distance from the position of the paddle shaft at the initiation of the pull phase to the value at the farthest point and was calculated for each axis. The catch and release positions of the paddle were the x-coordinates of the marker of the paddle shaft at the initiation and end of the pull phase, respectively. The catch and finish positions were normalized according to the position of the pelvic center. We defined the pelvic and torso rotation angles as the maximum angular difference around the vertical axis of the pelvic and upper body segments during the pull phase, respectively. The joint angle at catch was calculated for the external rotation, elevation, and horizontal abduction of the shoulder, elbow flexion, forearm pronation, and wrist flexion.

The integrated EMG of each muscle was normalized by their respective values obtained from the maximal voluntary contraction trials. The EMG data were divided into two halves during the pull phase: (1) the first half, which was the phase from the catch to peak force, and (2) the second half, which was the phase from the peak force to release.

As for the reproducibility of the analysis for major variables, the average intraindividual coefficient of variation was 4.9% for peak force, 2.1% for stroke rate, 2.6% for stroke length, 9.0% for pelvic rotation, 15.9% for torso rotation, 13.2% for latissimus dorsi EMG, and 7.3% for vastus lateralis EMG.

### Statistical Analyses

All data are presented as mean ± standard deviation. Based on the finding that novice skills are less reproducible (Limonta et al., 2010), seven strokes completed by the participant were assumed as independent variables in the present study. We analyzed the measured variables using repeated-measures two-way analysis of variance (time × stroke rate). If significant interactions were identified, the main effect of time was subsequently analyzed using Tukey's *post-hoc* test. The effect size was expressed as partial eta squared (η^2^), assuming a small, medium, and large effect size of <0.02, 0.02–0.26, and >0.26, respectively (Bakeman, 2005). Spearman's Rho correlation coefficients (*r*) were calculated. The significance level was set at *P* < 0.05.

## Results

### On-Water Test

The time required for paddling for 270 m was reduced by 7.3% from pre-training (99.3 s) to post-training (92.0 s).

### On-Ergometer Test

#### Paddling Kinetics

Peak force significantly decreased for medium (*P* < 0.001) and slow (*P* = 0.016) stroke rates after training (Table 1). After training, the impulse significantly increased in the x-direction for medium (*P* < 0.001) and slow (*P* < 0.001) stroke rates; conversely, the impulse significantly decreased in the y- direction for maximal (*P* < 0.001) and slow (*P* = 0.014) stroke rates and in the z-direction for all three stroke rates. The average impulse significantly increased in the x-direction for all three stroke rates after training (maximal and slow *P* < 0.001, medium *P* = 0.020).

**Table 1:** Effect of 4-week kayaking training on the paddling kinetics, paddling kinematics, and temporal parameters.

			**Time**		**Analysis of variance**				***Post-hoc* comparisons**
**Variable**	**Direction**	**Stroke rate**	**Pre**	**Post**	**Factor**	***F***	**Partial η2**	***P***	
Peak force (N kg^−1^)	X	Maximal	2.59 ± 0.09	2.63 ± 0.16	Time	18.56	0.34	<0.001^*^	
		Medium	2.16 ± 0.12	1.83 ± 0.06	Stroke rate	260.73	0.94	<0.001^*^	
		Slow	1.78 ± 0.09	1.63 ± 0.09	Time × Stroke rate	18.56	0.34	<0.001^#^	Medium, Slow
Impulse (N kg^−1^ s)	X	Maximal	137.4 ± 4.3	134.9 ± 4.0	Time	55.26	0.61	<0.001^*^	
		Medium	113.4 ± 1.6	120.2 ± 2.6	Stroke rate	127.46	0.88	<0.001^*^	
		Slow	109.3 ± 4.3	129.1 ± 3.3	Time × Stroke rate	35.69	0.66	<0.001^#^	Medium, Slow
	Y	Maximal	43.4 ± 2.4	28.9 ± 8.4	Time	26.76	0.43	<0.001^*^	
		Medium	32.8 ± 1.1	33.7 ± 3.5	Stroke rate	8.48	0.32	0.001^*^	
		Slow	32.7 ± 1.8	27.1 ± 2.0	Time × Stroke rate	12.85	0.42	<0.001^#^	Maximal, Slow
	Z	Maximal	20.6 ± 2.4	0.8 ± 12.7	Time	55.46	0.61	<0.001^*^	
		Medium	18.1 ± 2.2	10.6 ± 3.6	Stroke rate	4.08	0.18	0.025^*^	
		Slow	14.3 ± 2.7	2.2 ± 1.8	Time × Stroke rate	4.18	0.19	0.023^#^	Maximal, Medium, Slow
Average impulse (N kg^−1^ s^−1^)	X	Maximal	71.9 ± 1.9	91.1 ± 5.4	Time	169.26	0.82	<0.001^*^	
		Medium	61.3 ± 1.6	67.3 ± 2.3	Stroke rate	294.34	0.94	<0.001^*^	
		Slow	50.8 ± 1.8	60.3 ± 2.4	Time × Stroke rate	19.48	0.52	<0.001^#^	Maximal, Medium, Slow
	Y	Maximal	22.8 ± 1.6	19.4 ± 5.4	Time	4.22	0.10	0.047^*^	
		Medium	17.8 ± 1.6	18.8 ± 2.3	Stroke rate	28.76	0.62	<0.001^*^	
		Slow	15.2 ± 1.8	12.7 ± 2.4	Time × Stroke rate	3.08	0.15	0.059	
	Z	Maximal	10.8 ± 1.4	0.5 ± 8.4	Time	34.21	0.49	<0.001^*^	
		Medium	9.8 ± 1.1	5.9 ± 1.9	Stroke rate	4.27	0.19	0.022^*^	
		Slow	6.6 ± 1.2	1.0 ± 0.9	Time × Stroke rate	2.97	0.14	0.064	
Stroke rate (spm)		Maximal	62.8 ± 1.2	81.0 ± 2.9	Time	139.7	0.80	<0.001^*^	
		Medium	64.9 ± 1.7	67.2 ± 2.2	Stroke rate	254.64	0.93	<0.001^*^	
		Slow	55.8 ± 1.0	56.1 ± 1.8	Time × Stroke rate	92.85	0.84	<0.001^#^	Maximal, Medium
Stroke length (m)	X	Maximal	1.53 ± 0.04	1.71 ± 0.02	Time	136.02	0.79	<0.001^*^	
		Medium	1.30 ± 0.03	1.43 ± 0.05	Stroke rate	222.01	0.93	<0.001^*^	
		Slow	1.38 ± 0.04	1.44 ± 0.02	Time × Stroke rate	9.95	0.36	<0.001^#^	Maximal, Medium, Slow
	Y	Maximal	0.75 ± 0.02	0.50 ± 0.09	Time	22.41	0.38	<0.001^*^	
		Medium	0.53 ± 0.04	0.58 ± 0.03	Stroke rate	6.93	0.28	0.003^*^	
		Slow	0.61 ± 0.05	0.59 ± 0.02	Time × Stroke rate	34.66	0.66	<0.001^#^	Maximal, Medium
	Z	Maximal	−0.51 ± 0.05	−0.57 ± 0.18	Time	24	0.40	<0.001^*^	
		Medium	−0.57 ± 0.05	−0.76 ± 0.19	Stroke rate	9.78	0.35	<0.001^*^	
		Slow	−0.59 ± 0.07	−0.88 ± 0.08	Time × Stroke rate	3.1	0.15	0.057	
Pull time (s)		Maximal	0.90 ± 0.02	0.76 ± 0.04	Time	3.31	0.08	0.077	
		Medium	0.89 ± 0.02	0.88 ± 0.06	Stroke rate	173.63	0.91	<0.001^*^	
		Slow	1.05 ± 0.02	1.12 ± 0.05	Time × Stroke rate	26.79	0.60	<0.001^#^	
Transition time (s)		Maximal	0.04 ± 0.01	0.01 ± 0.01	Time	16.84	0.32	<0.001^*^	
		Medium	0.03 ± 0.01	0.04 ± 0.02	Stroke rate	9.18	0.34	0.001^*^	
		Slow	0.03 ± 0.01	0.00 ± 0.02	Time × Stroke rate	18.44	0.51	<0.001^#^	Maximal, Medium, Slow
Catch position (m)		Maximal	−0.88 ± 0.01	−1.04 ± 0.03	Time	913.54	0.96	<0.001^*^	
		Medium	−0.84 ± 0.01	−1.03 ± 0.01	Stroke rate	15.47	0.46	<0.001^*^	
		Slow	−0.91 ± 0.01	−1.02 ± 0.02	Time × Stroke rate	18.52	0.51	<0.001^#^	Maximal, Medium, Slow
Release position (m)		Maximal	0.69 ± 0.04	0.67 ± 0.02	Time	15.93	0.31	<0.001^*^	
		Medium	0.48 ± 0.03	0.42 ± 0.02	Stroke rate	288.13	0.94	<0.001^*^	
		Slow	0.49 ± 0.03	0.45 ± 0.01	Time × Stroke rate	1.88	0.09	0.167	

*Values in two colums on the left are means and standard deviation. X, anterior-posterior direction; Y, medial-lateral direction; Z, superior-inferior direction*.

*Time: Time factor (Pre vs. Post); Stroke rate: Stroke rate factor (Maximal vs. Medium vs. Slow)*.

* *P < 0.05 indicating a significant main effect*.

# *P < 0.05 indicating a significant interaction*.

*The stroke rate, which showed significant differeces in the intereaction and main effect of time, is noted in the most right column*.

#### Paddling Kinematics and Temporal Parameters

The stroke rate significantly increased for maximal (*P* < 0.001) and medium (*P* = 0.029) stroke rates after training (Table 1). The stroke length in the x-direction significantly increased for all three stroke rates after training (maximal and medium *P* < 0.001, slow *P* = 0.002). The stroke length in the y-direction significantly decreased for the maximal (*P* < 0.001) stroke rate after training, whereas the opposite occurred for the medium (*P* = 0.047) stroke rate. The transition time for all three stroke rates significantly decreased after training (maximal and slow *P* < 0.001, medium *P* = 0.018). However, the pull time was not significantly (*P* = 0.077) changed after training. There was a significant increase in the paddle catch position and elbow position after four-week training for all three stroke rates (all *P* < 0.001).

#### Body Kinematics

The torso rotation angular displacement significantly increased for the slow stroke rate after training (Table 2). The shoulder's external rotation angle in the catch position significantly increased for the maximal (*P* = 0.029) and slow (*P* < 0.001) stroke rates after training. Moreover, the shoulder's horizontal abduction angle significantly increased for all three stroke rates after training (all *P* < 0.001). After training, the wrist flexion angle significantly increased (medium *P* = 0.012, slow *P* < 0.001).

**Table 2:** Effect of 4-week kayaking training on the joint angle at catch position, pelvic and torso rotation angular displacements.

		**Mean** **±** **SD**		**Analysis of variance**				***Post-hoc* comparisons**
**Variable**	**Stroke rate**	**Pre**	**Post**	**Factor**	***F***	**Partial η2**	***P***	
Shoulder external rotation	Maximal	−30.7 ± 2.5	−27.2 ± 3.6	Time	11.39	0.24	0.002^*^	
	Medium	−33.0 ± 1.0	−33.3 ± 3.1	Stroke rate	7.09	0.28	0.003^*^	
	Slow	−33.8 ± 2.8	−27.9 ± 2.6	Time × Stroke rate	3.99	0.18	0.027^#^	Maximal, Slow
Shoulder elevation	Maximal	27.8 ± 2.2	42.6 ± 2.3	Time	213.99	0.86	<0.001^*^	
	Medium	28.3 ± 2.4	40.7 ± 4.8	Stroke rate	10.77	0.37	<0.001^*^	
	Slow	31.2 ± 2.6	48.4 ± 3.1	Time × Stroke rate	1.86	0.09	0.170	
Shoulder horizontal abduction	Maximal	76.0 ± 2.4	90.3 ± 5.6	Time	291.91	0.89	<0.001^*^	
	Medium	77.7 ± 1.9	97.7 ± 3.2	Stroke rate	8.12	0.31	0.001^*^	
	Slow	77.4 ± 2.6	97.8 ± 2.0	Time × Stroke rate	3.36	0.16	0.046^#^	Maximal, Medium, Slow
Elbow flexion	Maximal	126.9 ± 1.9	129.9 ± 3.4	Time	2.83	0.07	0.101	
	Medium	123.4 ± 1.8	119.0 ± 3.7	Stroke rate	25.26	0.58	<0.001^*^	
	Slow	122.7 ± 2.7	119.3 ± 3.3	Time × Stroke rate	5.79	0.24	0.007^#^	
Forearm pronation	Maximal	−29.1 ± 3.1	−42.2 ± 4.8	Time	90.08	0.71	<0.001^*^	
	Medium	−27.6 ± 3.2	−41.5 ± 6.5	Stroke rate	12.1	0.40	<0.001^*^	
	Slow	−21.7 ± 2.4	−34.2 ± 3.6	Time × Stroke rate	0.09	0.00	0.918	
Wrist flexion	Maximal	33.0 ± 2.7	32.8 ± 3.2	Time	11.32	0.24	0.002^*^	
	Medium	30.3 ± 2.8	33.9 ± 4.0	Stroke rate	0.45	0.02	0.642	
	Slow	28.7 ± 2.0	35.0 ± 2.3	Time × Stroke rate	3.84	0.18	0.031^#^	Medium, Slow
Pelvic	Maximal	4.47 ± 1.37	0.31 ± 3.37	Time	90.92	0.72	<0.001^*^	
	Medium	4.87 ± 1.82	−1.45 ± 1.3	Stroke rate	0.99	0.05	0.382	
	Slow	4.73 ± 1.38	−1.98 ± 1.61	Time × Stroke rate	1.75	0.09	0.188	
Torso	Maximal	−0.44 ± 8.16	1.39 ± 13.39	Time	0.53	0.01	0.469	
	Medium	5.53 ± 9.05	−0.38 ± 3.56	Stroke rate	0.21	0.01	0.811	
	Slow	2.46 ± 3.7	0.76 ± 9.16	Time × Stroke rate	0.72	0.04	0.493	
Pelvic rotation angular displacement	Maximal	26.1 ± 2.3	29.4 ± 2.0	Time	21.11	0.37	<0.001^*^	
	Medium	18.1 ± 1.8	19.6 ± 0.7	Stroke rate	158.23	0.90	<0.001^*^	
	Slow	16.6 ± 1.3	18.7 ± 1.1	Time × Stroke rate	1.02	0.05	0.370	
Torso rotation anglular displacement	Maximal	86.2 ± 5.3	77.9 ± 4.1	Time	4.71	0.12	0.037^*^	
	Medium	63.1 ± 2.5	66.5 ± 2.2	Stroke rate	37.15	0.67	<0.001^*^	
	Slow	46.5 ± 17.4	67.3 ± 3.8	Time × Stroke rate	12.07	0.40	<0.001^#^	Slow

*Values in two colums on the left are means and standard deviation. Units are degree. Shoulder external rotation; internal(+)/external(-), Shoulder elevation; elevation(+)/depression(-), Shoulder horizontal abduction; adduction(+)/abduction(-), Elbow flexion; extension(+)/flexion(-), Forearm pronation; pronation(+)/supination(-), Wrist flexion; flexion(+)/extension(-)*.

*Time: Time factor (Pre vs. Post); Stroke rate: Stroke rate factor (Maximal vs. Medium vs. Slow)*.

* *P < 0.05 indicating a significant main effect*.

# *P < 0.05 indicating a significant interaction*.

*The stroke rate, which showed significant differeces in the intereaction and main effect of time, is noted in the most right column*.

#### EMG

During the first half of the pull phase, which mainly involved kayak propulsive velocity, a significant increase in EMG activity was observed in the triceps brachii (maximal stroke rate, *P* < 0.001), latissimus dorsi (all three stroke rates, *P* < 0.001), and vastus lateralis (maximal stroke rate, *P* < 0.001), whereas a decrease was detected in the anterior deltoid (maximal stroke rate, *P* < 0.001), trapezius (all three stroke rates, *P* < 0.001), and musculus obliquus externus abdominis (all three stroke rates, maximal and medium *P* < 0.001, slow *P* = 0.023) (Table 3).

**Table 3:** Effect of 4-week kayaking training on the EMG activities for each muscle at 1st and 2nd halves of pull phase.

			**Mean** **±** **SD**		**Analysis of variance**				***Post-hoc* comparisons**
**Muscle**	**Phase**	**Stroke rate**	**Pre**	**Post**	**Factor**	***F***	**Partial η2**	***P***	
Biceps brachii	1st half	Maximal	6.6 ± 1.2	10.2 ± 3.0	Time	34.66	0.49	<0.001^*^	
		Medium	7.0 ± 0.9	8.6 ± 1.8	Stroke rate	1.3	0.07	0.286	
		Slow	5.6 ± 0.8	9.3 ± 0.6	Time × Stroke rate	1.85	0.09	0.171	
Triceps brachii		Maximal	8.8 ± 1.3	18.4 ± 3.9	Time	32.65	0.48	<0.001^*^	
		Medium	6.4 ± 0.7	6.9 ± 0.9	Stroke rate	81.93	0.82	<0.001^*^	
		Slow	6.0 ± 0.8	5.4 ± 0.5	Time × Stroke rate	34.45	0.66	<0.001^#^	Maximal
Anterior deltoid		Maximal	23.2 ± 2.8	5.2 ± 1.4	Time	36.57	0.50	<0.001^*^	
		Medium	17.5 ± 4.4	13.8 ± 6.6	Stroke rate	23.28	0.56	<0.001^*^	
		Slow	22.9 ± 2.6	23.6 ± 2.4	Time × Stroke rate	23.62	0.57	<0.001^#^	Maximal
Trapezius		Maximal	41.8 ± 6.2	31.5 ± 6.5	Time	157.38	0.81	<0.001^*^	
		Medium	35.5 ± 3.5	15.6 ± 1.3	Stroke rate	33.69	0.65	<0.001^*^	
		Slow	34.7 ± 4.2	13.8 ± 1.8	Time × Stroke rate	6.31	0.26	0.005^#^	Maximal, Medium, Slow
Latissimus dorsi		Maximal	16.8 ± 2.9	65.9 ± 11.2	Time	440.75	0.92	<0.001^*^	
		Medium	15.4 ± 1.1	43.5 ± 3.6	Stroke rate	31.48	0.64	<0.001^*^	
		Slow	15.6 ± 2.3	38.3 ± 1.9	Time × Stroke rate	25.81	0.59	<0.001^#^	Maximal, Medium, Slow
Vastus lateralis		Maximal	36.1 ± 1.8	84.4 ± 13.6	Time	60.94	0.63	<0.001^*^	
		Medium	24.3 ± 1.7	19.0 ± 1.3	Stroke rate	241.5	0.93	<0.001^*^	
		Slow	16.8 ± 1.7	15.5 ± 2.0	Time × Stroke rate	93.38	0.84	<0.001^#^	Maximal
Musculus obliquus externus abdominis		Maximal	97.5 ± 27.0	41.5 ± 7.6	Time	70.74	0.66	<0.001^*^	
		Medium	50.0 ± 11.3	22.1 ± 6.2	Stroke rate	33.93	0.65	<0.001^*^	
		Slow	41.8 ± 5.4	25.4 ± 3.7	Time × Stroke rate	8.74	0.33	<0.001^#^	Maximal, Medium, Slow
Biceps brachii	2nd half	Maximal	6.2 ± 1.2	58.4 ± 8.7	Time	450.88	0.93	<0.001^*^	
		Medium	8.7 ± 1.6	29.2 ± 5.0	Stroke rate	90.7	0.83	<0.001^*^	
		Slow	4.2 ± 0.6	16.7 ± 2.9	Time × Stroke rate	81.98	0.82	<0.001^#^	Maximal, Medium, Slow
Triceps brachii		Maximal	19.6 ± 2.3	25.1 ± 2.7	Time	70.92	0.66	<0.001^*^	
		Medium	10.7 ± 1.3	15.7 ± 1.3	Stroke rate	138.21	0.88	<0.001^*^	
		Slow	10.8 ± 0.7	14.0 ± 2.6	Time × Stroke rate	1.77	0.09	0.184	
Anterior deltoid		Maximal	38.7 ± 5.0	40.1 ± 6.6	Time	16.67	0.32	<0.001^*^	
		Medium	23.4 ± 2.3	19.9 ± 2.6	Stroke rate	133.75	0.88	<0.001^*^	
		Slow	23.0 ± 1.4	10.4 ± 2.6	Time × Stroke rate	11.86	0.40	<0.001^#^	Slow
Trapezius		Maximal	73.5 ± 2.9	48.2 ± 3.8	Time	619.68	0.95	<0.001^*^	
		Medium	52.9 ± 3.4	37.3 ± 2.0	Stroke rate	139.9	0.89	<0.001^*^	
		Slow	57.9 ± 2.5	30.3 ± 2.9	Time × Stroke rate	16.28	0.47	<0.001^#^	Maximal, Medium, Slow
Latissimus dorsi		Maximal	43.2 ± 6.9	52.2 ± 2.9	Time	11.17	0.24	0.002^*^	
		Medium	30.4 ± 2.9	38.2 ± 3.3	Stroke rate	59.1	0.77	<0.001^*^	
		Slow	32.9 ± 4.2	29.4 ± 4.0	Time × Stroke rate	9.07	0.33	<0.001^#^	Maximal, Medium
Vastus lateralis		Maximal	36.2 ± 5.4	69.4 ± 6.9	Time	26.23	0.42	<0.001^*^	
		Medium	18.5 ± 0.6	10.1 ± 1.1	Stroke rate	508.59	0.97	<0.001^*^	
		Slow	16.8 ± 1.9	9.7 ± 1.1	Time × Stroke rate	139.36	0.89	<0.001^#^	Maximal, Medium, Slow
Musculus obliquus externus abdominis		Maximal	27.9 ± 5.7	39.7 ± 4.7	Time	52.22	0.59	<0.001^*^	
		Medium	13.7 ± 2.5	20.3 ± 2.1	Stroke rate	110.03	0.86	<0.001^*^	
		Slow	11.6 ± 3.1	18.0 ± 2.8	Time × Stroke rate	2.44	0.12	0.101	

*Values in two colums on the left are means and standard deviation. Units are Electromyography (EMG) in % maximal voluntary contraction EMG*.

*Time: Time factor (Pre vs. Post); Stroke rate: Stroke rate factor (Maximal vs. Medium vs. Slow)*.

* *P < 0.05 indicating a significant main effect*.

# *P < 0.05 indicating a significant interaction*.

*The stroke rate, which showed significant differeces in the intereaction and main effect of time, is noted in the most right column*.

**Figure 1:**
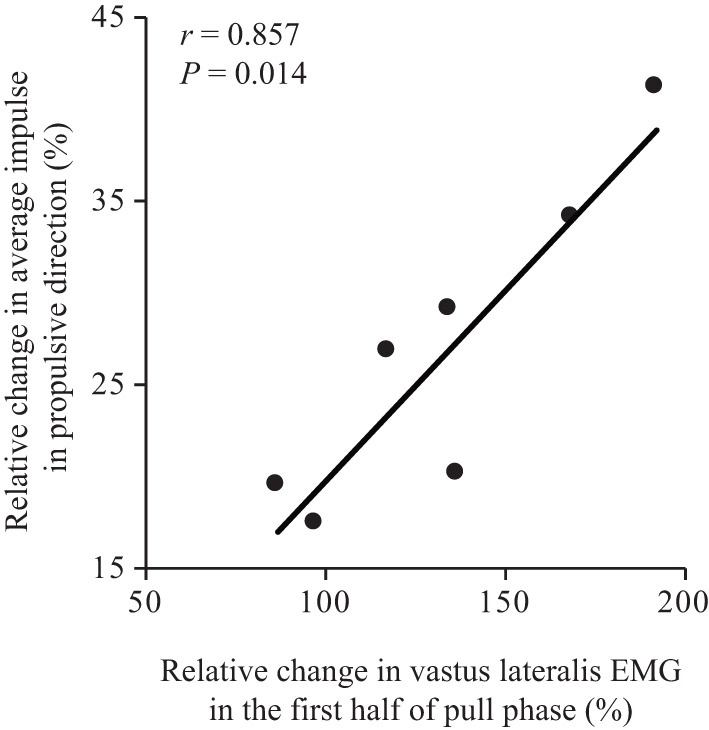
Relationship between relative change in propulsive average impulse and vastus lateralis EMG, with four-week kayak training. The regression line is shown because the relationship was found to be significant.

With respect to the results of correlation analysis, the relative change in average impulse in the x-direction with training exhibited a significant correlation with the relative change in vastus lateralis EMG during the first half of the pull phase with training (*r* = 0.857, *P* = 0.014) (Figure 1).

## Discussion

After four-week kayak training, the time of on-water all-out paddling for 270 m improved by ~7%. The present case study has shown for the first time that even a novice paddler can improve the on-water average kayak velocity (which was calculated as the distance divided by the time and increased from 9.79 km/h to 10.57 km/h) after four-week kayak training. Thus, the following question arises: What mechanism is involved in increasing average kayak velocity? It is known that a significant relationship between average kayak velocity and stroke rate exists (Hay and Yanai, 1996; Bourgois et al., 1998; Brown et al., 2011). A deterministic model based on a review of previous literature revealed that the stroke rate was strongly correlated with the average horizontal kayak velocity (McDonnell et al., 2013). In the present study, the stroke rate at maximal effort was significantly greater in post-training than in pre-training (Table 1), indicating that a higher stroke rate would contribute to an increase in average kayak velocity after four-week training. The stroke rate was inversely proportional to the stroke time (stroke rate = 1/stroke time), and the stroke time was composed of times of pull, transition, and return phases. It should be noted that the phase terminology varied in the previous literature (McDonnell et al., 2012). In the present study, we defined the pull phase from the catch of one side to the release, the transition phase from the release to the catch of the opposite side, and the return phase from the release to the catch of one side. Hay and Yanai (Brown et al., 2011) reported a significant correlation (*r* = −0.83, *P* < 0.05) between the average water phase time (synonymous with pull time in this study) and average kayak velocity for a group of 10 elite paddlers, indicating that shorter water phase times were significantly associated with higher average kayak velocity. Our results showed that the substantial pull time, which defined as pull time relative to the stroke displacement, was probably shorter in post-training than in pre-training because of the lengthening stroke length in the anterior-posterior direction after training. On the other hand, a shortened transition time (Table 1) may be involved in increasing the displacement of transition and return phases, given the deceleration of the kayak during the transition and return phases, and hence likely lead to faster average kayak velocities.

Another potential mechanism involves an increase in average impulse along the propulsive direction. This is the first study to measure and separate the average impulse in three orthogonal directions. Millward (1987) and Michael et al. (2012) suggested that the maintenance of force near the peak throughout the water phase (synonymous with pull phase in this study) is of greater importance to performance than the peak force itself, as the force pattern can vary from almost triangular to rectangular (Hill, 2002) with the same peak force. This idea is consistent with the results of peak force in the present study (Table 1). Gomes et al. (2015) showed that the relationship between impulse and mean velocity in each stroke rate (60, 80 spm, and race pace) were highly significant (60 spm, *r* = 0.888; 80 spm, *r* = 0.896; race pace, *r* = 0.847; all *P* < 0.01), indicating that, if the area under the force-time curve is increased and the stroke rate maintained, the average kayak velocity should increase. We calculated the impulse as well as average impulse to account for the varying paddling times. The results for paddling kinetics revealed that the impulse and average impulse were more than three to six times higher in the anterior-posterior direction, which is the main direction of kayak propulsion, than in the lateral-medial and superior-inferior directions during paddling at all three stroke rates. In addition, during maximal paddling effort, the impulse in propulsive direction was not changed while the average impulse in propulsive direction significantly increased from pre to post-training. With regards to the percentage of change with training, the average impulse in propulsive direction was positively correlated with vastus lateralis EMG in the early pull phase (Figure 1). These correlations indicate that training induced an increase in average impulse in propulsive direction was caused by knee extension. Vastus lateralis EMG in the early pull phase was also significantly greater in post-training than in pre-training during maximal paddling (Table 3). Indeed, leg movement during paddling has gained interest in recent times. Nilsson and Rosdahl (2016) reported that while paddling at a higher intensity, restricting leg movement can reduce paddle force and kayak speed by 21% and 16%, respectively. Therefore, paddlers frequently use their legs while paddling at higher intensities. In contrast, it may be difficult to acquire greater pelvic and torso rotations during maximal paddling with only four weeks of kayak training. During the pull phase initiation, paddlers sit in a position of torso flexion, and the pelvis is rotated away from the pulling paddle. During the pull phase, paddlers remain seated in a torso flexion position, and the pelvis rotates ~100° to the ipsilateral side of the paddle (Bjerkefors et al., 2018). Limonta et al. (2010). reported that intermediate-level and elite paddlers display more accentuated pelvic movements in the horizontal plane than novice paddlers. In the present study, there was no significant difference in pelvic and torso rotations during maximal paddling between pre-training and post-training, which does not support our hypothesis. Thus, for novice paddlers, coaches should give specific instructions to increase pelvic rotations.

It is unclear whether the training-induced increase in stroke length in the anterior-posterior direction was related to the increase in velocity. The increase in the catch position after training was associated with an increase in the stroke length. During maximal paddling, a simultaneous decrease in the shoulder's external rotation angle and an increase in the shoulder's horizontal adduction angle in the catch position after training enabled the paddle to be placed in a more forward position. Although McDonnell et al. (2013) reported no significant correlation between stroke displacement and velocity for grouped data, one case of a male paddler showed shorter stroke displacement in testing protocols with faster average kayak velocities (Brown et al., 2011). Lengthening stroke length was primarily attributed to longer displacement of transition and return phases as well as higher average kayak velocity; however, no direct correlations were reported (Baker et al., 1999). Hence, there exist no sufficient data to determine a relationship or general trend between stroke length and average kayak velocity.

The small sample size, lower reproducibility, and measurement environment are potential limitations of our study. We tested limited samples regarding the type and number of participants and the number of stroke cycles. Further systematic examinations should involve longitudinal studies and include participants at different performance levels. This study presents variability among paddling kinetics and kinematics, body kinematics, and EMG (CV: 2.1–15.9%). Lower reproducibility probably resulted from variations in each segment position and produced action potentials of each muscle during paddling. From a biomechanical perspective, the fact that ergometers test on a solid surface presents major differences in lateral stability and stroke rate between paddling on the ergometer and on-water (Klitgaard et al., 2020).

In conclusion, after four-week kayak training for a novice paddler, the reduction in the time required for on-water paddling for 270 m was associated with (1) increased average impulse along the propulsive direction caused by an increase in vastus lateralis EMG and (2) a higher stroke rate, which was attributed to a reduction in pull and transition times. Based on the findings of this case study, coaches and athletes may have a better understanding of how to improve the skills of novice paddlers.

## Data Availability Statement

The original contributions presented in the study are included in the article/Supplementary Material, further inquiries can be directed to the corresponding author/s.

## Ethics Statement

The studies involving human participants were reviewed and approved by The Human Research Ethics Committee at Kanagawa University. The patients/participants provided their written informed consent to participate in this study.

## Author Contributions

RK contributed to the conception and design of the study. RK and SK performed data collection. SK analyzed the data and performed statistical analyses. KE contributed to material preparation. RK wrote the first draft of the manuscript. SK wrote sections of the manuscript. RK and SK contributed to manuscript revision, read, and all authors approved the submitted version.

## Conflict of Interest

The authors declare that the research was conducted in the absence of any commercial or financial relationships that could be construed as a potential conflict of interest.

## Publisher's Note

All claims expressed in this article are solely those of the authors and do not necessarily represent those of their affiliated organizations, or those of the publisher, the editors and the reviewers. Any product that may be evaluated in this article, or claim that may be made by its manufacturer, is not guaranteed or endorsed by the publisher.
