# Supplementary material for: Effects of Four-Week Kayak Training on Three-Dimensional Paddling Kinetics, Body Kinematics, and Electromyography Activity in a Novice Paddler: A Case Study
Source: Front Sports Act Living. 2021 Jul 27;3:694989. doi: 10.3389/fspor.2021.694989 (PMC8353118; doi:10.3389/fspor.2021.694989)
Supplement: Supplementary file 1 [file Data_Sheet_1.DOCX]

Supplementary Material

# Supplementary Methods

# 1.1 Training

The training was conducted for four weeks, which consisted of practicing kayak paddling on the river using a surfski apparatus (V5, Epic Kayaks). The training program was summarized in supplemental table 1. If the training was deemed dangerous because of strong wind or waves, an air-braked kayak ergometer (K1 ergo, Australian Sports Commission) was used instead. The training program was finalised by the participant himself, taking into account the advice of the instructor (co-author), former elite kayak paddlers (international level), weather conditions, and his own physical condition. The diet during the training period was similar to that before the training period.

# 1.2 Experimental design

The participant was required to perform all-out paddling for 270 m on the river for the on-water test. The ergometer test required the participant to perform all-out paddling using the kayak ergometer. The paddling on-ergometer test lasted approximately 20 s. Within this time, the participant was capable of completing at least seven strokes at the following stroke rates: 56 strokes per minute (spm; defined as slow), 69 spm (defined as medium), and maximal. The participant was free to select the maximal stroke rate. However, he had to perform paddling to the maximum possible extent. We used an audible metronome (ME-D1, Yamaha, Shizuoka, Japan) in the trials of 56 spm and 69 spm. The order of the stroke rates was randomised. A recovery time was 1 min between the trials. For each stroke rate, the participant was provided a preparation time of 10 s to match the stroking pace to the target stroke rate. The temperature and humidity settings in the laboratory were similar before and after the training.

# 1.3 EMG

Electromyography (EMG) recordings were captured from seven muscles on the dominant side of the body. We used standard wireless electrodes (Trigo, Delsys) to record the muscle activity from the skin over the biceps brachii, triceps brachii, anterior deltoid, upper trapezius, latissimus dorsi, vastus lateralis, and transverse abdominal muscles. The reason these muscles were chosen is that the major muscle groups for the paddling motion are the arms, shoulders, back, trunk, and thighs, based on the EMG data of previous studies (Fleming 2012a, 2012b; Trevithick 2007). All skin was carefully abraded and cleaned with alcohol before electrode placement. The electrode placement for each muscle was based on the position and procedures described by the Surface EMG for Non-invasive Assessment of Muscles (SENIAM) recommendations (Freriks et al. 1999). Therefore, the position of the electrode was similar before and after the training. The EMG signals were amplified (×100) using a standard biosignal recording system and band-pass filtered at 20-450 Hz before the sampling.

The EMG data were full-wave rectified digitally and smoothed by applying a bidirectional second-order Butterworth low-pass filter. The cut-off frequency was set to 5 Hz. The integrated EMG of each muscle was normalized by their respective values obtained from the maximal voluntary contraction trials, performed on each day of the measurement, before and after the training. The EMG data was time-interpolated over a time base, with 200 points for each stroke cycle. The EMG data was divided into two halves during the pull phase. The phase from the catch to the peak force was defined as the first half, and that from the peak force to release was defined as the second half.

# 1.4 Motion capture and load cell

A strain gauge load cell (LUR-A-1KNSA1, Kyowa Electronic Instruments) was placed between the rope from the ergometer flywheel and the right end of the paddle shaft. The force was collected during paddling at a sampling rate of 1500 Hz. We performed the calibration statically by loading the calibrated weights.

A motion-capture system (Raptor-12, Motion Analysis, California, United States of America) was used to measure the three-dimensional coordinates of reflective markers during the on-ergometer paddling test. Reflective markers were attached to the jugular notch, the xiphoid process of the sternum, second sacral vertebra, seventh cervical vertebra, ninth thoracic vertebra, second sacral vertebra, acromions, anterior superior iliac spine, greater trochanter, lateral malleolus, medial malleolus, medial epicondyle, lateral epicondyle, radial styloid process, ulnar styloid process, second metacarpal bone, and fifth metacarpal bone. One marker was also attached to each paddle shaft. Four additional markers were attached to each side of the transducers. We defined the x, y, and z axes in the global coordinate system as the posterior (+) / anterior (-), lateral (+) / medial (-), and superior (+) / inferior (-) directions, respectively. The frame rate of the motion-capture system was 150 Hz.

The marker data were smoothed by applying a bidirectional second-order Butterworth low-pass filter. We conducted a residual analysis to calculate the cut-off frequency for each marker (Winter 2009). The force data were smoothed by applying a bidirectional second-order Butterworth low-pass filter twice. The cut-off frequencies were 200 Hz and were calculated for each marker by residual analysis (Winter 2009). The paddle kinetics and kinematics, body kinematics were time-interpolated over a time base, with 200 points for each stroke cycle.

The centre of the circumcircle made by three of the four markers attached to the load cell was defined as the load cell's center. We calculated a vector normal to the plane through the markers and along the long axis of the shaft in the global coordinate system at each frame.

# 1.5 Body composition

We measured the body weight, the percentage of body fat, the amount of muscle, and lean body mass using a body composition analyser (MC-190, Tanita).

Reference

- Fleming N, Donne B, Fletcher D. Effect of kayak ergometer elastic tension on upper limb EMG activity and 3D kinematic. J Sports Sci Med (2012a) 11:430–437.
- Fleming N, Donne B, Fletcher D, Mahony N. A biomechanical assessment of ergometer task specificity in elite flat-water kayakers. J Sports Sci Med (2012b) 11:16–25.
- Freriks B, Hermens H, Disselhorst-Klug C, Rau G. The recommendations for sensors and sensor placement procedures for surface electromyography. In: Hermens HJ, Freriks B, Merletti R, et al. (eds) European Recommendations for Surface Electromyography. Enschede: Roessingh Research and Development (1999). p. 15–54.
- Trevithick BA, Ginn KA, Halaki M, Balnave R. Shoulder muscle recruitment patterns during a kayak stroke performed on a paddling ergometer. J Electromyogr Kinesiol (2007) 17:74–79.
- Winter DA. Processing of raw kinematic data. In: Winter DA (ed) Biomechanics and Motor Control of Human Movement. Hoboken: John Wiley & Sons (2009). p. 64–74.

# Supplementary Results

The participant’s body weight decreased from 59.7 kg to 57.9 kg and percentage of body fat reduced from 14.1% to 12.7% post-training. Moreover, lean body mass changed from 51.3 kg to 50.6 kg. However, muscle mass remained nearly constant (48.6 kg pre-training vs. 47.9 kg post-training).

# Supplementary Figures and Tables

## Supplementary Figures

**Supplementary Figure 1. One stroke cycle was divided into three phases; pull, transition, and return phases.** The pull phase defined as the phase from 1 (i.e., minimum value of the x-coordinate of the right edge marker of the paddle shaft) to 2 (i.e., the maximum value of the y-coordinate of the right edge marker of the paddle shaft) while the transition phase defined as the phase from 2 to 3 (i.e., maximum value of the y-coordinate of the left edge marker of the paddle shaft). The return phase defined as the phase from 2 to 4 (i.e., minimum value of the x-coordinate of the right edge marker of the paddle shaft in next stroke cycle).

**Supplementary Figure 2.** **Mean force-time curves, pre- (white) and post-training (grey).** Data are divided into the x- (anterior-posterior, left panel), y- (medial-lateral, middle panel), and z-directions (superior-inferior, right panel) during paddling at slow (bottom row), medium (middle row), and maximal (top row) stroke rates. The 0% x-axis value represents the beginning of the pull phase on the ipsilateral side (i.e., the beginning of the right pull phase for the right side), while 100% represents the return to the beginning of the pull phase on the ipsilateral side (i.e., one stroke cycle). The vertical solid line represents the end of the pull phase. The vertical dashed line represents the beginning of the transition phase. Values are presented as mean (thick solid line) and standard deviation (shadow).

**Supplementary Figure 3. Mean pelvic and torso angular displacements-time curves, pre- (white) and post-training (grey).** Data are presented for the pelvic (left panel) and torso (right panel) angular displacements during paddling at slow (bottom row), medium (middle row), and maximal (top row) stroke rates. The 0% x-axis value represents the beginning of the pull phase on the ipsilateral side (i.e. the beginning of the right pull phase for the right side), while 100% represents the return to the beginning of the pull phase on the ipsilateral side (i.e. one stroke cycle). The vertical solid line represents the end of the pull phase. The vertical dashed line represents the beginning of the transition phase. Values are presented as mean (thick solid line) and standard deviation (shadow).

## Supplementary Table
